# New proposal involving nanoformulated melatonin targeted to the mitochondria as a potential COVID-19 treatment

**DOI:** 10.2217/nnm-2020-0371

**Published:** 2020-12-03

**Authors:** Virna Margarita Martín Giménez, Natalia Prado, Emiliano Diez, Walter Manucha, Russel J Reiter

**Affiliations:** ^1^Instituto de Investigaciones en Ciencias Químicas, Facultad de Ciencias Químicas y Tecnológicas, Universidad Católica de Cuyo, San Juan, Argentina; ^2^Área de Farmacología, Departamento de Patología, Facultad de Ciencias Médicas, Universidad Nacional de Cuyo, Mendoza, Argentina; ^3^Instituto de Medicina y Biología Experimental de Cuyo (IMBECU), Consejo Nacional de Investigaciones Científicas y Tecnológicas (CONICET), Mendoza, Argentina; ^4^Instituto de Fisiología, Facultad de Ciencias Médicas, Universidad Nacional de Cuyo, Mendoza, Argentina; ^5^Department of Cell Systems & Anatomy, UT Health San Antonio Long School of Medicine, San Antonio, TX, USA

**Keywords:** acute respiratory distress syndrome, cytokine storm, edema, mitochondrial dysfunction, nanoformulations, SARS-CoV-2, viral infection

Melatonin may be a powerful auxiliary therapy in the prevention and treatment of viral infections, such as coronavirus disease 2019 (COVID-19) [1]. Casually, the so-called ‘super immunity’ of bats and the usually high levels of melatonin in children could contribute to their high resistance to the SARS-CoV-2 virus [2]. Melatonin seems to play a key role in suppressing COVID-19 infections. This endogenous antioxidant inhibits cell apoptosis, blocks the inflammasomes that mediate lung inflammation, reduces blood vessel permeability which limits alveolar edema, improves anxiety and sleeps habits that stimulate general immunity and prevents lung fibrosis [3]. These complications, which are usually the main consequences of COVID-19, may be significantly attenuated by melatonin [4].

Melatonin theoretically provokes a switch from reactive to quiescent phenotypes in immune cells, through a shift in their metabolism from glycolytic metabolism to oxidative phosphorylation. Moreover, melatonin inhibits neutrophil recruitment. Furthermore, a SARS-CoV-2 infection suppresses mitochondrial melatonin production by inducing cytosolic glycolytic metabolism which deprives the mitochondria of acetyl-co-enzyme A, a necessary cosubstrate for melatonin synthesis; the loss of mitochondrial melatonin contributes to the typical ‘cytokine storm’ observed in COVID-19 infection. The glycolytic metabolism that causes mitochondrial dysfunction is likely reversed by melatonin [5]. In addition to its mitochondrial protective effects, melatonin has also important immunomodulatory, anti-inflammatory and antiviral properties, which have been documented in multiple experimental models of lung diseases [6]. Melatonin also downregulates the expression of matrix metallopeptidase 9, which is involved with the immunoinflammatory response mediated by neutrophils and the methoxyindole modulates the function of angiotensin converting enzyme 2, the main receptor that allows the entrance of SARS-CoV-2 into the cells [7]. It has also been suggested that the administration of high doses of melatonin may attenuate the exacerbated neuroinflammation caused by SARS-CoV-2, which would lessen the brain damage observed in many patients with COVID-19 [8]. A recent study examined the structural and physicochemical features of melatonin with the aid of different electronic structure and molecular-mechanics methods. The electronic properties of melatonin allow predictions of its bio-activity. In this study, it was reported that melatonin has potent activity against SARS-CoV-2 proteins. The collective results strongly suggest that melatonin would be highly useful in the mitigation of COVID-19 severity [9].

Noteworthy is that COVID-19 mortality rates increase with age of the patient. Aging is known to be related to an increased mitochondrial dysfunction and elevated oxidative damage accompanied by a drop in melatonin production. Since melatonin is an important antioxidant, melatonin supplementation in the elderly would be potentially beneficial in the prevention of mortality due to COVID-19 [10]. Additionally, melatonin not only directly exerts important actions against COVID-19, it may also synergistically potentiate the anti-inflammatory actions of other endogenous substances, such as vitamin D, which also seems to be beneficial in the prevention and treatment of this viral infection [11]. In other clinical conditions that co-exist with a COVID-19 infection. In other words, diabetics, obesity and cardiovascular disease, melatonin has been suggested as an adjuvant in the therapeutic protocols to improve clinical outcomes in these patients with increased risk of mortality [12].

The exogenous administration of melatonin does, however, have some issues. One of the main physicochemical and pharmacokinetic limitations of this substance is its short half-life in the physiological microenvironment after its administration due to its high susceptibility to oxidative degradation [13]. Another limitation with regard to the conventional administration of melatonin may be its high lipophilicity, which negatively influences its oral bio-availability [14].

Therapeutic nanoformulations offer several advantages over conventional pharmaceutical preparations since drug release kinetics are modulated by the choice of nanomaterials with specific features. The use of nanoformulations also allows programming of the drug release site in response to a remote or site-specific trigger, such as changes in pH, temperature or enzymatic activity [15]. Moreover, nanoformulations allow greater efficacy of therapies. This is because nanomaterials have a high surface area to volume ratio, which provides a greater contact surface with the physiological medium, favoring the rapid dissolution of poorly water-soluble drugs. Furthermore, the stability of molecules contained in the nanoformulations is greater than that of drugs in free form. This is because the nanostructures act as protective coatings against different destabilizing factors, such as pH, enzymes, hydrolysis, etc.; this also results in prolonged pharmacological effects [16]. In addition, therapeutic nanoformulations usually have less toxicity than conventional pharmaceutical formulations. This happens because drug nanocarriers may be directed to specific target sites using pharmacological targeting strategies (active, passive and/or physical targeting), thereby avoiding or reducing side effects associated to systemic drug distribution. Moreover, nanoformulations require significantly less drug than conventional formulations, which reduces their toxicity [17]. With specific regard to melatonin, its mitochondrial targeting would represent an attractive therapeutic goal. Melatonin’s side effects are generally not be a limitation for its exogenous administration, since this substance has a high safety profile and are considered an especially innocuous drug [4].

Several nanoplatforms for the dermal delivery of melatonin were developed in the last several years; these include ethosomes, liposomes, niosomes, solid lipid nanoparticles, polymeric nanoparticles and cyclodextrins. The use of these nanoplatforms for melatonin delivery as an antioxidant agent would improve its efficacy in comparison with conventional melatonin formulations due to its greater protection from premature oxidation and the enhancement in cell uptake and bio-availability [18]. Cationic solid lipid nanoparticle carriers of melatonin have been developed to potentiate the ocular hypotensive effect of this drug. This nanoformulation not only caused a significant reduction in intraocular pressure but also had good tolerability on the ocular surface [19]. Nanoformulated melatonin also proved to be more effective in the protection against genotoxicity induced by etoposide in the HepG2 cell line compared with conventionally administered melatonin. The reduction of both reactive oxygen species production and DNA damage, and the increase in intracellular glutathione concentrations in this cultured cell line was more significant with the use of nanoformulated melatonin compared with solubilized melatonin added to the incubation medium [20]. Moreover, it has also been demonstrated that the protective effect of melatonin incorporated into polymeric nanoparticles on adipose-derived mesenchymal stem cells cultured under oxidative stress conditions was better than that of conventional melatonin [13]. One of the most advanced and complex nanosystems developed for melatonin delivery is based on ‘smart nanocarriers’. Thus, biosmart nanoparticles which concentrate in mitochondria and repair tissue damaged by ischemia have been designed. These core/shell nanoparticles are composed of the two layers. The core is loaded with melatonin and the shell is loaded with circular DNA. At the acute stage of ischemia, the pro-oxidative cell microenvironment stimulates the release of melatonin from these biosmart nanoparticles and prevents apoptosis induced by reactive oxygen species. At the chronic stage of ischemia, circular DNA senses hypoxia and produces VEGF for inducing revascularization of ischemic tissue [21].

On the basis of the technology available, melatonin delivery nanosystems should be examined with the aim of improving the stability, selectivity and efficacy of melatonin to create new therapeutic alternatives for the treatment of COVID-19 and/or other viral infections.

## References

[1] Naveen Kumar SK, Hemshekhar M, Jagadish S Melatonin restores neutrophil functions and prevents apoptosis amid dysfunctional glutathione redox system. J. Pineal Res. 69(3), e12676 (2020). 32597503 10.1111/jpi.12676

[2] Shneider A, Kudriavtsev A, Vakhrusheva A Can melatonin reduce the severity of COVID-19 pandemic? Int. Rev. Immunol. 39(4), 153–162 (2020). 32347747 10.1080/08830185.2020.1756284

[3] García JA, Volt H, Venegas C Disruption of the NF-κB/NLRP3 connection by melatonin requires retinoid-related orphan receptor-alpha and blocks the septic response in mice. FASEB J. 29, 3863–3875 (2015). 26045547 10.1096/fj.15-273656

[4] Zhang R, Wang X, Ni L COVID-19: melatonin as a potential adjuvant treatment. Life Sci. 250, 117583 (2020). 32217117 10.1016/j.lfs.2020.117583 PMC7102583

[5] Reiter RJ, Sharma R, Ma Q Melatonin inhibits COVID-19-induced cytokine storm by reversing aerobic glycolysis in immune cells: a mechanistic analysis. Med. Drug Discov. 6, 100044 (2020). 32395713 10.1016/j.medidd.2020.100044 PMC7211589

[6] Anderson G, Reiter RJ Melatonin: roles in influenza, COVID-19, and other viral infections. Rev. Med. Virol. 30(3), e2109 (2020). 32314850 10.1002/rmv.2109 PMC7235470

[7] Hazra S, Chaudhuri AG, Tiwary BK Matrix metallopeptidase 9 as a host protein target of chloroquine and melatonin for immunoregulation in COVID-19: a network-based meta-analysis. Life Sci. 257, 118096 (2020). 32679150 10.1016/j.lfs.2020.118096 PMC7361122

[8] Romero A, Ramos E, López-Muñoz F Coronavirus disease 2019 (COVID-19) and its neuroinvasive capacity: is it time for melatonin? Cell. Mol. Neurobiol. (2020) (Epub ahead of print). 10.1007/s10571-020-00938-8 PMC7415199 32772307

[9] Al-Zaqri N, Pooventhiran T, Alsalme A Structural and physico-chemical evaluation of melatonin and its solution-state excited properties, with emphasis on its binding with novel coronavirus proteins. J. Mol. Liq. 318, 114082 (2020). 32863490 10.1016/j.molliq.2020.114082 PMC7443329

[10] Özturk G, Akbulut KG, Guney Ş Melatonin, aging and COVID-19: could melatonin be beneficial for COVID-19 treatment in elderly? Turk. J. Med. Sci. (2020) (Epub ahead of print). 10.3906/sag-2005-356 PMC7605095 32777902

[11] Martín Giménez VM, Inserra F, Tajer CD Lungs as target of COVID-19 infection: protective common molecular mechanisms of vitamin D and melatonin as a new potential synergistic treatment. Life Sci. 254, 117808 (2020). 32422305 10.1016/j.lfs.2020.117808 PMC7227533

[12] El-Missiry MA, El-Missiry ZMA, Othman AI Melatonin is a potential adjuvant to improve clinical outcomes in individuals with obesity and diabetes with coexistence of COVID-19. Eur. J. Pharmacol. 882, 173329 (2020). 32615182 10.1016/j.ejphar.2020.173329 PMC7324339

[13] Ma Q, Yang J, Huang X Poly(lactide-co-glycolide)-monomethoxy-Poly-(polyethylene glycol) nanoparticles loaded with melatonin protect adipose-derived stem cells transplanted in infarcted heart tissue. Stem Cells 36(4), 540–550 (2018). 29327399 10.1002/stem.2777

[14] DeMuro RL, Nafziger AN, Blask DE The absolute bioavailability of oral melatonin. J. Clin. Pharmacol. 40(7), 781–784 (2000). 10883420 10.1177/00912700022009422

[15] Giménez VMM, Camargo AB, Kassuha D Nanotechnological strategies as smart ways for diagnosis and treatment of the atherosclerosis. Curr. Pharm. Des. 24(39), 4681–4684 (2018). 30636583 10.2174/1381612825666190110154609

[16] Giménez VM, Sperandeo N, Faudone S Preparation and characterization of bosentan monohydrate/ε-polycaprolactone nanoparticles obtained by electrospraying. Biotechnol. Prog. 35(2), e2748 (2019). 30548149 10.1002/btpr.2748

[17] Giménez VMM, Fuentes LB, Kassuha DE Current drug nano-targeting strategies for improvement in the diagnosis and treatment of prevalent pathologies such as cardiovascular and renal diseases. Curr. Drug Targets 20(14), 1496–1504 (2019). 31267869 10.2174/1389450120666190702162533

[18] Milan AS, Campmany ACC, Naveros BC Antioxidant nanoplatforms for dermal delivery: melatonin. Curr. Drug Metab. 18(5), 437–453 (2017). 28228077 10.2174/1389200218666170222145908

[19] Leonardi A, Bucolo C, Drago F Cationic solid lipid nanoparticles enhance ocular hypotensive effect of melatonin in rabbit. Int. J. Pharm. 478(1), 180–186 (2015). 25448580 10.1016/j.ijpharm.2014.11.032

[20] Shokrzadeh M, Ghassemi-Barghi N Melatonin loading chitosan-tripolyphosphate nanoparticles: application in attenuating etoposide-induced genotoxicity in HepG2 cells. Pharmacology 102(1–2), 74–80 (2018). 29940567 10.1159/000489667

[21] Lin Y, Liu J, Bai R Mitochondria inspired nanoparticles with microenvironment-adapting capacities for on-demand drug delivery after ischemic injury. ACS Nano 14(9), 11846–11859 (2020). 32880428 10.1021/acsnano.0c04727

